# Systematic Review on Antidepressant Use and Bleeding Risk After Dental Extractions: Evidence Gaps and Clinical Implications

**DOI:** 10.3390/jcm14176140

**Published:** 2025-08-30

**Authors:** Alexander Nemeth, Kayvon Rahimi, Sachin Karande, Tea Romasco, Adam Lowenstein, Rodrigo dos Santos Pereira, Carlos Fernando Mourão

**Affiliations:** 1 Department of Basic and Clinical Translational Sciences, School of Dentistry, Tufts University, Boston, MA 02111, USA; 2 Department of Oral & Maxillofacial Surgery, University of Grande Rio—UNIGRANRIO, Rio de Janeiro 25071-202, Brazil; 3 Department of Medical, Oral and Biotechnological Sciences, “G. D’Annunzio” University of Chieti-Pescara, 66100 Chieti, Italy; 4 Department of Oral & Maxillofacial Surgery, Rio de Janeiro Federal University, Rio de Janeiro 21941-617, Brazil

**Keywords:** antidepressants, bleeding, dental extraction, hemostasis, serotonin reuptake inhibitors, systematic review

## Abstract

**Background/Objectives:** This review aimed to evaluate whether patients undergoing dental extractions while taking antidepressants experience increased intra-operative or post-operative bleeding compared to patients not taking these medications. **Methods:** A comprehensive literature search was conducted across PubMed, EMBASE, Web of Science, Scopus, and ClinicalTrials.gov for randomized controlled trials (RCTs) published before 17 August 2025. Studies were included if they compared bleeding outcomes between antidepressant users and non-users undergoing dental extraction procedures. The review followed the Preferred Reporting Items for Systematic Reviews and Meta-Analyses (PRISMA) guidelines and was registered with the International Prospective Register of Systematic Reviews (PROSPERO, CRD42025645035). **Results:** Of 689 studies screened, no RCTs met the eligibility criteria. Only one retrospective study, which did not match the inclusion criteria, identified a 1% incidence of bleeding complications in users of selective serotonin reuptake inhibitors (SSRIs) undergoing invasive dental procedures. However, it lacked a control group and standardized methodology, so this study was included in the discussion section. **Conclusions:** The lack of high-quality evidence, especially studies examining dynamic coagulation parameters like bleeding time and prothrombin time before and after antidepressant use, highlights a significant gap in the research. These findings emphasize the urgent need for well-designed clinical trials to determine the potential effect of antidepressants on bleeding risk.

## 1. Introduction

Antidepressants, including selective serotonin reuptake inhibitors (SSRIs), serotonin–norepinephrine reuptake inhibitors (SNRIs), tricyclic antidepressants (TCAs), monoamine oxidase inhibitors (MAOIs), and newer atypical agents such as bupropion, represent a pharmacological mainstay for treating depressive and anxiety disorders and are among the most prescribed medications worldwide [1]. In the United States alone, approximately 13.6% of adults over the age of 20 are taking prescribed antidepressants [2]. Overall, antidepressant use has risen globally over recent decades, with SSRIs being among the most frequently prescribed types. This trend indicates increased awareness and treatment of depressive and anxiety disorders globally [3,4].

SSRIs such as fluoxetine, sertraline, and citalopram are generally well tolerated but are frequently associated with gastrointestinal symptoms, sexual dysfunction, insomnia, and other early- and late-emerging adverse effects [5,6]. SNRIs, including venlafaxine and duloxetine, share many SSRI side effects and may additionally contribute to hypertension, tachycardia, and increased bleeding tendencies in susceptible patients [5]. TCAs, such as amitriptyline and nortriptyline, have prominent anticholinergic effects, orthostatic hypotension, weight gain, and cardiotoxicity, particularly in overdoses, which limits their tolerability [7,8]. MAOIs, including phenelzine and tranylcypromine, are less frequently used due to dietary restrictions and potential hypertensive crises, as well as serious drug–drug interactions, necessitating cautious patient selection [9,10]. Bupropion, the most common of the atypical antidepressants, has significant side effects as well, including dry mouth, nausea, increased sweating, tremors, seizures, hypertension, and anxiety [11].

Although TCAs and MAOIs can influence serotonergic neurotransmission, they do so via distinct mechanisms. TCAs may partially inhibit serotonin and norepinephrine reuptake, whereas MAOIs increase extracellular serotonin levels indirectly by inhibiting the monoamine oxidase enzyme, not the serotonin transporter (5-HTT). Therefore, their potential impact on platelet function and bleeding risk may differ from that of SSRIs and SNRIs [1].

Moreover, these antidepressant medications, especially those that act on serotonergic pathways, have been associated with increased bleeding tendencies and increased risk of major bleeding events [12]. Specifically, they have been found to have an increased incidence of gastrointestinal bleeding and excessive intra-operative bleeding across various surgical contexts. Multiple case–control studies have reported an elevated risk of abnormal bleeding during spinal surgery among patients using antidepressants [13,14]. Similarly, SSRIs have been associated with an increased risk of intra-operative bleeding in cosmetic breast surgery, as well as a greater likelihood of re-operation due to post-operative hemorrhage in breast cancer patients [15,16]. In orthopedic surgery, serotonergic antidepressant users were found to have nearly a fourfold increased risk of requiring blood transfusions compared to those taking non-serotonergic antidepressants or no antidepressants at all [17]. Comparable findings have been reported in patients undergoing hip replacement surgery [18]. Notably, however, one case–control study examining postpartum hemorrhage found no significant difference in bleeding risk between users of SSRIs and those taking other types of antidepressants, suggesting that the impact of these medications on bleeding may vary by surgical context [19].

**Figure 1:**
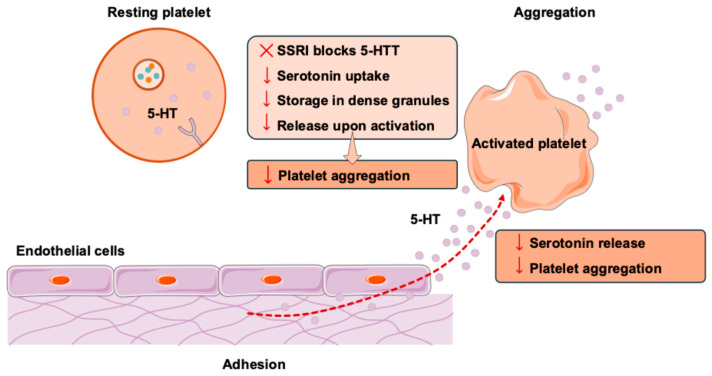
Role of selective serotonin reuptake inhibitors (SSRIs) in platelet function and aggregation. This figure illustrates platelet adhesion, activation, and aggregation at a site of vascular injury. Serotonin (5-HT), taken up by 5-HT transporters (5-HTT), is released upon activation and promotes vasoconstriction and platelet aggregation via 5-HT_2_A receptors. SSRIs block 5-HTT, reducing intraplatelet serotonin levels and impairing platelet aggregation, potentially increasing bleeding risk.

In oral surgery, the management of bleeding is a critical concern, as the feasibility and safety of many procedures are influenced by intra-operative and post-operative bleeding. However, the implications of antidepressant use on bleeding outcomes remain unclear [20,21]. As a result, there is a significant gap in the literature regarding the perioperative bleeding risk for this population in dental contexts [22,23,24]. Similar exclusion practices have been observed in randomized controlled trials of arthroscopic rotator cuff surgery, abdominal hysterectomy, and prospective observational studies following elective spinal surgery [25,26,27,28]. The mechanisms underlying this association are still being investigated; however, it is widely believed that serotonergic antidepressants inhibit platelet aggregation by depleting intraplatelet serotonin, an essential component of clot formation. Figure 1 provides a graphical description of this process.

This effect is further exacerbated when patients simultaneously use non-steroidal anti-inflammatory drugs (NSAIDs), which are routinely prescribed for dental pain management before and after extractions. The combination of SSRIs and NSAIDs significantly increases the risk of bleeding compared to either drug alone [29,30]. Although bleeding risks associated with TCAs and MAOIs are less well characterized than those associated with SSRIs and SNRIs, their anticholinergic and vasoactive properties may still influence hemostasis and wound healing in invasive procedures [7,9]. As an outcome of these results, surgeons across healthcare often confer with the patient’s psychiatrist about whether antidepressants should be stopped prior to any surgery.

Surgical procedures like dental extractions are commonly performed on patients taking antidepressants, and dentists routinely treat individuals on long-term antidepressants without clear data on hemorrhagic risk. Until robust evidence emerges, clinicians should perform individualized bleeding risk assessments, coordinate care with prescribing physicians, and employ meticulous local hemostatic techniques when managing these patients. Since serotonergic antidepressants have been linked to higher bleeding risks in surgeries such as orthopedic, spinal, and breast cancer procedures, this systematic review examined whether these risks also occur during dental extractions. The review analyzed clinical evidence and patient outcomes to determine if antidepressant use is associated with increased perioperative bleeding risk in dental surgeries.

## 2. Materials and Methods

The protocol for this systematic review was developed in accordance with the Preferred Reporting Items for Systematic Reviews and Meta-Analyses (PRISMA) guidelines and was prospectively registered in the International Prospective Register of Systematic Reviews (PROSPERO) database (registration number: CRD42025645035).

### 2.1. Focused Question and Outcome Definition

In patients undergoing dental extraction procedures, do patients undergoing concurrent antidepressant drug therapy have an increased risk of bleeding compared to patients who are not undergoing antidepressant drug therapy?

Clinically relevant post-operative bleeding was defined as any bleeding event requiring active intervention (e.g., re-treatment, surgical hemostasis, tamponade), leading to unplanned re-consultation or delayed discharge, or associated with prolonged hemostasis or the need for blood transfusion. This operational definition was guided by bleeding classification frameworks like the Bleeding Academic Research Consortium (BARC) and adapted to capture bleeding events with a clear clinical impact in dental surgical settings.

### 2.2. Eligibility Criteria

Criteria for inclusion were established using the Patients, Intervention, Comparison, Outcome, and Study (PICOS) design and were as follows:

P: Patients undergoing dental extraction procedures;
I: Patients taking antidepressants;
C: Patients not taking antidepressants;
O: Bleeding;
S: Randomized clinical trials.

There were no restrictions on the publication date. The criteria for excluding articles were as follows: (i) studies that were not randomized clinical trials and (ii) studies published in non-English languages.

### 2.3. Search Strategy

A comprehensive electronic literature search was conducted to identify relevant studies published prior to 17 August 2025. The databases searched included PubMed/MEDLINE, Embase, Web of Science, and Scopus. To capture gray literature, we additionally searched ClinicalTrials.gov. Gray literature was limited exclusively to this trial registry; other sources, such as dissertations, conference abstracts, preprints, or institutional reports, were not included. Reference lists of all included studies were manually screened to identify any additional eligible publications. The search strategy applied the following terms and Boolean operators:

(((antidepressants) OR (SSRI)) OR (SNRI)) OR (wellbutrin)) AND ((bleeding) OR (hemorrhage) OR (bleeding events)) AND (dental OR extractions OR tooth).

Full search strategies for each database are provided in Table 1.

### 2.4. Study Selection and Data Extraction

First, the studies were screened through an analysis of titles and abstracts. Studies that met the eligibility criteria were selected for full review to verify their adherence to the eligibility criteria. This was completed by one researcher (A.N.) and verified by a second researcher (C.M.).

Data extraction was conducted by A.N. and reviewed by C.M. Any disagreements were resolved by careful discussion. The extracted data included authors (year of publication), patient conditions, treatment groups (antidepressant drugs used), number of subjects (sex, age), measures of bleeding and bleeding outcomes, any other adverse complications or outcomes measured, type of dental extraction, and follow-up. If any relevant data were missing, the reviewers planned to contact the original study authors to obtain the needed information for this systematic review.

## 3. Results

A qualitative synthesis of the search outcomes was performed. Due to the heterogeneity of study designs and reported outcomes, as well as the complete absence of randomized clinical trials meeting the eligibility criteria, a meta-analysis could not be conducted.

**Figure 2:**
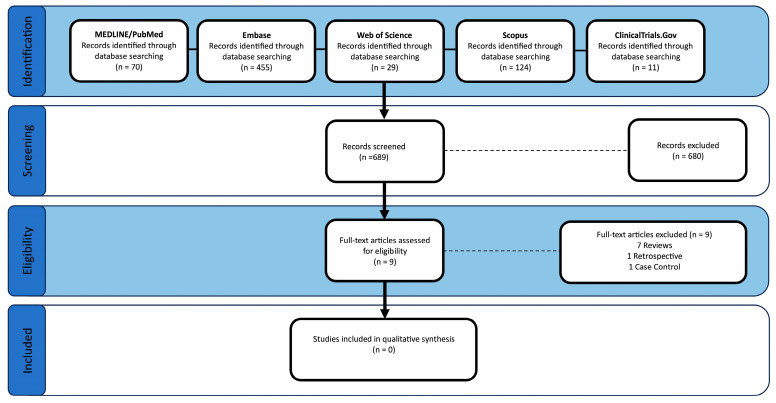
Preferred Reporting Items for Systematic Reviews and Meta-Analyses (PRISMA) study flow diagram.

The initial search produced 689 studies (70 from MEDLINE/PubMed, 455 from Embase, 29 from Web of Science, 124 from Scopus, and 11 from ClinicalTrials.gov). ClinicalTrials.gov was searched to identify unpublished trials; other forms of gray literature, such as preprints, dissertations, and conference proceedings, were not screened. After removal of duplicates and screening of titles and abstracts, nine articles were selected for full-text analysis. However, all nine were excluded due to study design limitations, including seven narrative reviews, one retrospective cohort study, and one case–control study. Thus, no studies were selected for inclusion in this review. The search flow is illustrated in Figure 2 (PRISMA Flow Diagram).

Although no randomized trials were identified, the screening process revealed several notable patterns. Many studies discussed bleeding risks in surgical or pharmacologic contexts not directly related to dental procedures, while others involving dental surgery frequently excluded patients using antidepressants. Additionally, most studies lacked standardized definitions for bleeding outcomes, failed to report the severity or timing of hemorrhagic events, and did not consistently document the use of concurrent medications such as non-steroidal anti-inflammatory drugs or anticoagulants.

Significant heterogeneity was also observed in the types and dosages of antidepressants reported, the surgical protocols employed, and the hemostatic strategies used. These methodological inconsistencies and reporting gaps not only precluded inclusion in the systematic review but also reflect a broader lack of structured, prospective research addressing this clinically relevant issue.

The absence of eligible randomized studies itself represents an important finding, highlighting the need for future research with robust methodology to assess perioperative bleeding risk in patients undergoing antidepressant therapy during dental extractions.

## 4. Discussion

This systematic review aimed to investigate whether patients taking antidepressant medications undergoing dental extractions experience an increase in intra- and/or post-operative bleeding compared to patients not taking antidepressants. Despite a comprehensive search across multiple databases, including gray literature, no randomized controlled trials (RCTs) met the inclusion criteria. The absence of RCTs underscores a significant gap in the dental literature regarding the perioperative bleeding risk associated with antidepressant use in dental procedures, a question with increasing relevance given the high and growing prevalence of antidepressant use worldwide.

While no studies met the eligibility criteria for inclusion in this review, one retrospective study addressed the current question. Napeñas et al. [27] conducted a retrospective cohort study at the Carolinas Medical Center dental clinic, assessing the risk of oral bleeding complications in patients taking SSRIs, specifically citalopram, escitalopram, fluoxetine, fluvoxamine maleate, paroxetine, sertraline, and dapoxetine, following invasive dental procedures. They reported on 92 patients using SSRIs, with a mean age of 51.2 years, consisting of 77% females and 23% males. These patients underwent a total of 145 invasive procedures, with more than 75% involving extractions. Among these procedures, the authors identified two cases of post-operative bleeding complications, representing approximately 1%. While the study lacked control or comparison groups, the authors concluded that the risk of post-operative bleeding in patients on antidepressants following invasive dental procedures is low to negligible.

However, this limited evidence, along with the exclusion of antidepressant users in numerous clinical studies involving dental extractions, as observed in trials by Toledano-Serrabona et al., Li et al., and Steenen et al. [22,23,24], reflects a broader concern among investigators regarding the potential confounding effects of these medications on clinical outcomes. Such exclusion criteria may stem from findings in the broader surgical literature, which have linked antidepressant use, particularly SSRIs, to increased bleeding risks across various types of procedures, including orthopedic, spinal, breast, and abdominal surgeries, along with a general increased risk of bleeding, notably in the gastrointestinal tract [13,14,15,16,17,18,19].

These associations are biologically plausible, possibly explaining the tendency of clinicians to generalize the findings across different surgical fields. Serotonin (5-HT) plays two key roles in blood clotting, vasoconstriction, and platelet aggregation. After a vascular injury, platelets adhere to the exposed subendothelial components and collagen, causing platelet activation. Platelet activation involves shape change and release of granules, including serotonin, adenosine diphosphate (ADP), and thromboxane A2. For the purpose of the present discussion, the focus was given to the role of 5-HT in this step. Platelet-released 5-HT causes local vasoconstriction, which helps slow and reduce blood flow to decrease blood loss and promote the formation of a stable clot [31]. Additionally, 5-HT released by platelets enhances fibrin formation and induces a thrombogenic state in the bloodstream [32]. 5-HT also plays an important role in amplifying the platelet response by binding to the 5-HT_2_A receptor on the platelet plasma membrane, promoting further platelet activation and a positive feedback mechanism. Platelets, however, are unable to synthesize their 5-HT and rely on 5-HT transporters (5-HTT) on their plasma membranes to take up 5-HT from the blood plasma for storage in granules and later release in blood clotting. SSRIs block 5-HTT, thus decreasing platelet intracellular 5-HT levels [33]. In theory, this decrease in platelet intracellular 5-HT levels leads to an impaired blood-clotting response (Figure 1).

In addition to their individual effects on hemostasis, antidepressants have been shown to increase the bleeding risk when used in combination with other medications that disrupt hemostasis, particularly NSAIDs and anticoagulants such as warfarin. In their systematic review and meta-analysis [34], Weng and Lan found treatment with both a direct oral anticoagulant (DOAC), such as direct thrombin inhibitors (i.e., dabigatran) and factor Xa inhibitors (i.e., rivaroxaban), and SSRIs or SNRIs were associated with a significantly higher risk of major bleeding. In their study of hospitalized patients, Quinn et al. [35] found that there is an increased bleeding risk of 30–70% in hospitalized patients taking a combination of SSRIs and vitamin K antagonists, such as coumadin. In their meta-analysis, Anglin et al. [36] found that while SSRIs alone were associated with an increased risk of bleeding, the risk was significantly elevated when SSRIs were taken alongside NSAIDs. This risk should not be taken lightly in the dental setting, as NSAIDs are commonly prescribed for pre- and post-operative pain control in oral surgeries and dental extractions.

Interestingly, while these concerns are reflected in the general surgical fields, evidence specific to dentistry remains sparse. The only identified study directly addressing this question was a retrospective cohort study by Napeñas et al. [37], which reported a post-operative bleeding rate of approximately 1% among SSRIs users undergoing invasive dental procedures. Although the study lacked a control group and therefore was limited in its ability to establish causality or comparative risk, it suggests that the absolute risk of bleeding in this population may be lower than expected. However, the small sample size, retrospective design, lack of standardization in the procedure types, and hemostatic protocol warrant caution when extrapolating these findings to broader clinical contexts.

In contrast to major surgeries, where blood loss is more substantial and transfusions are common, the localized and relatively low vascular nature of many dental procedures may mitigate the bleeding risk in antidepressant users. This may explain the low complication rate reported by Napeñas et al. [37] and raises essential questions about the generalizability of data from orthopedic or oncologic surgeries to the dental setting.

Moreover, the heterogeneity of antidepressant classes adds further complexity. While SSRIs and SNRIs have demonstrated antiplatelet effects, the effects of other antidepressants, such as TCAs, bupropion, or MAOIs, remain poorly defined. It is worth noting, however, that while TCAs may partially inhibit serotonin reuptake, and MAOIs increase extracellular serotonin levels via monoamine oxidase inhibition, their mechanisms differ from those of SSRIs and SNRIs. As such, any potential effects on platelet function and hemostasis remain theoretical and have not been conclusively demonstrated [7,8,9,10]. Nevertheless, the variable pharmacodynamics of these medications and potential integration with analgesics or sedatives may influence bleeding risk and recovery in ways that have not yet been systematically studied.

The lack of RCTs may also reflect the ethical and logistical challenges in conducting placebo-controlled trials involving antidepressant discontinuation in surgical patients. Withholding antidepressants from individuals with major depressive or anxiety disorders carries the risk of withdrawal, relapse, or worsening of psychiatric symptoms, which must be carefully weighed against the hypothetical risk of increased bleeding. This ethical dilemma underscores the importance of observational or pragmatic trial designs in evaluating real-world outcomes without compromising patient safety.

Given the increasing number of patients undergoing dental procedures while on chronic antidepressant therapy, high-quality prospective research is urgently needed. Such studies should aim to stratify bleeding risk by antidepressant class and dosage, control for concurrent medications, such as NSAIDs or anticoagulants, standardize definitions and measurements of bleeding complications, and report outcomes across various types of dental extractions and oral surgeries. Until such evidence is available, clinical decision-making should be individualized, balancing psychiatric stability with surgical safety. Dentists and oral surgeons should consider consulting with a patient’s primary care provider prior to surgery, especially in complex cases or when other risk factors for bleeding are present. Additionally, proactively employing intra-operative and post-operative bleeding control strategies is recommended for patients taking serotonergic agents.

This systematic review has several limitations that should be considered when interpreting its findings. The most important limitation is the lack of RCTs that meet the eligibility criteria, which prevented a quantitative analysis and weakened the strength of the conclusions. As a result, the review was unable to assess causality or determine the bleeding risk associated with antidepressant use during dental extractions. These limitations highlight the need for high-quality RCTs to better understand the actual effect of antidepressant therapy on perioperative bleeding in dental surgery.

Although randomized controlled trials offer the highest level of evidence, their feasibility in this clinical context is limited due to ethical concerns, particularly those related to the interruption of antidepressant therapy. Alternative study designs such as prospective cohorts with propensity score adjustment, treatment interruption analyses in real-world datasets, or pragmatic clinical trials conducted within routine care settings may provide more appropriate and ethically viable methods to evaluate perioperative bleeding risks. It is also crucial that future studies account for treatment duration, as long-term use of antidepressants may have cumulative effects on platelet function and hemostasis. Stratifying bleeding outcomes by exposure time would improve the clinical interpretation of findings.

Furthermore, although this review did not find any randomized clinical trials that met the eligibility criteria, it remains a valid and essential systematic review. According to the Cochrane Handbook, ‘empty reviews’ can serve as important indicators, pointing out where high-quality evidence is missing despite clinical significance [38]. While observational studies are frequently used to examine safety outcomes, including them would have introduced significant variability in study design, exposure assessment, and outcome definitions. Future research should focus on pragmatic randomized trials or carefully adjusted observational methods (e.g., propensity score matching) to assess bleeding risk in real-world dental care without affecting psychiatric stability.

## 5. Conclusions

This systematic review aimed to determine whether randomized clinical trials exist to evaluate the bleeding risks associated with antidepressant use in patients undergoing dental extractions. The results revealed a significant gap in the literature, with no eligible randomized trials identified. Although mechanistic concerns based on extrapolation from other surgical specialties suggest a plausible risk, definitive evidence within dentistry remains lacking.

Future research is urgently needed and should be designed to address current knowledge gaps through well-structured methodologies. An ideal approach would involve prospective cohort studies or pragmatic clinical trials with adequate sample sizes, stratified by antidepressant class, dosage, duration of use, and concurrent medications such as NSAIDs or anticoagulants. Additionally, because some antidepressants may be obtained over the counter or used irregularly in certain countries, future studies should also account for variability in usage patterns. These factors may influence platelet function and bleeding risk in ways that differ from chronic, stable use, and considering them would improve the clinical applicability of future findings.

These strategies will enable the generation of higher-quality and clinically applicable evidence to guide safe and personalized surgical decision-making for patients undergoing antidepressant therapy.

## Figures and Tables

**Table 1:** Search strategies for each database.

Database	Search Strategy
PubMed/MEDLINE	(“antidepressent”[All Fields] OR “antidepression”[All Fields] OR “antidepressive agents”[Pharmacological Action] OR “antidepressive agents”[Supplementary Concept] OR “antidepressive agents”[All Fields] OR “antidepressant”[All Fields] OR “antidepressive agents”[MeSH Terms] OR (“antidepressive”[All Fields] AND “agents”[All Fields]) OR “antidepressants”[All Fields] OR “antidepressive”[All Fields] OR “antidepressives”[All Fields] OR “SSRI ^1^”[All Fields] OR (“serotonin and noradrenaline reuptake inhibitors”[Pharmacological Action] OR “serotonin and noradrenaline reuptake inhibitors”[Supplementary Concept] OR “serotonin and noradrenaline reuptake inhibitors”[All Fields] OR “SNRI ^2^”[All Fields] OR “serotonin and noradrenaline reuptake inhibitors”[MeSH Terms] OR (“serotonin”[All Fields] AND “noradrenaline”[All Fields] AND “reuptake”[All Fields] AND “inhibitors”[All Fields])) OR (“bupropion”[Supplementary Concept] OR “bupropion”[All Fields] OR “amfebutamone”[All Fields] OR “bupropion”[MeSH Terms] OR “wellbutrin”[All Fields] OR “bupropion s”[All Fields] OR “bupropione”[All Fields])) AND (“bleedings”[All Fields] OR “hemorrhage”[MeSH Terms] OR “hemorrhage”[All Fields] OR “bleed”[All Fields] OR “bleeding”[All Fields] OR “bleeds”[All Fields] OR “bleeding event”[All Fields]) AND (“dental health services”[MeSH Terms] OR (“dental”[All Fields] AND “health”[All Fields] AND “services”[All Fields]) OR “dental health services”[All Fields] OR “dental”[All Fields] OR “dentally”[All Fields] OR “dentals”[All Fields] OR (“extract”[All Fields] OR “extract s”[All Fields] OR “extractabilities”[All Fields] OR “extractability”[All Fields] OR “extractable”[All Fields] OR “extractables”[All Fields] OR “extractant”[All Fields] OR “extractants”[All Fields] OR “extracted”[All Fields] OR “extractibility”[All Fields] OR “extractible”[All Fields] OR “extracting”[All Fields] OR “extraction”[All Fields] OR “extractions”[All Fields] OR “extractive”[All Fields] OR “extractives”[All Fields] OR “extracts”[All Fields]) OR (“teeth s”[All Fields] OR “teeths”[All Fields] OR “tooth”[MeSH Terms] OR “tooth”[All Fields] OR “teeth”[All Fields] OR “tooth s”[All Fields] OR “tooths”[All Fields]))
EMBASE	(‘antidepressants’/exp OR antidepressants OR ‘ssri’/exp OR ssri OR ‘snri’/exp OR snri OR ‘wellbutrin’/exp OR wellbutrin) AND (‘bleeding’/exp OR bleeding OR ‘hemorrhage’/exp OR hemorrhage OR ‘bleeding events’/exp OR ‘bleeding events’) AND (‘dental’/exp OR dental OR extractions OR ‘tooth’/exp OR tooth)
Web of Science	(((((antidepressants) OR (SSRI)) OR (SNRI)) OR (wellbutrin)) AND (bleeding OR hemorrhage OR bleeding events) AND (dental OR extractions OR tooth))
Scopus	TITLE-ABS-KEY((antidepressants OR ssri OR snri OR wellbutrin) AND (bleeding OR hemorrhage OR (bleeding events)) AND (dental OR extractions OR tooth))
ClinicalTrials.gov	((((antidepressants) OR (SSRI)) OR (SNRI)) OR (wellbutrin)) AND (bleeding OR hemorrhage OR (bleeding events)) AND (dental OR extractions OR tooth)

^1^ selective serotonin reuptake inhibitors; ^2^ serotonin–norepinephrine reuptake inhibitors.

## Data Availability

The data supporting the findings of this systematic review are available in the PROSPERO database (registration number: CRD42025645035).

## Abbreviations

The following abbreviations are used in this manuscript:

SSRIs	Serotonin reuptake inhibitors
SNRIs	Serotonin–norepinephrine reuptake inhibitors
TCAs	Tricyclic antidepressants
MAOIs	Monoamine oxidase inhibitors
5-HT	Serotonin
5-HTT	5-HT transporters
NSAIDs	Non-steroidal anti-inflammatory drugs
PRISMA	Preferred Reporting Items for Systematic Reviews and Meta-Analyses
PROSPERO	International Prospective Register of Systematic Reviews
PICOS	Patients, Intervention, Comparison, Outcome, and Study design
RCTs	Randomized controlled trials
ADP	Adenosine diphosphate
DOAC	Direct oral anticoagulant
